# A Simple and Effective Method for High Quality Co-Extraction of Genomic DNA and Total RNA from Low Biomass *Ectocarpus siliculosus*, the Model Brown Alga

**DOI:** 10.1371/journal.pone.0096470

**Published:** 2014-05-27

**Authors:** Maria Greco, Claudio A. Sáez, Murray T. Brown, Maria Beatrice Bitonti

**Affiliations:** 1 Department of Biology, Ecology and Earth Sciences, Laboratory of Plant Cyto-physiology, University of Calabria, Arcavacata di Rende (Cosenza), Italy; 2 School of Marine Sciences and Engineering, Plymouth University, Drake Circus, Plymouth, United Kingdom; 3 Departamento de Biología, Facultad de Química y Biología, Universidad de Santiago de Chile, Santiago, Chile; Deutsches Krebsforschungszentrum, Germany

## Abstract

The brown seaweed *Ectocarpus siliculosus* is an emerging model species distributed worldwide in temperate coastal ecosystems. Over 1500 strains of *E. siliculosus* are available in culture from a broad range of geographic locations and ecological niches. To elucidate the molecular mechanisms underlying its capacity to cope with different environmental and biotic stressors, genomic and transcriptomic studies are necessary; this requires the co-isolation of genomic DNA and total RNA. In brown algae, extraction of nucleic acids is hindered by high concentrations of secondary metabolites that co-precipitate with nucleic acids. Here, we propose a reliable, rapid and cost-effective procedure for the co-isolation of high-quality nucleic acids using small quantities of biomass (25-, 50- and 100 mg) from strains of *E. siliculosus* (RHO12; LIA4A; EC524 and REP10–11) isolated from sites with different environmental conditions. The procedure employs a high pH extraction buffer (pH 9.5) which contains 100 mM Tris-HCl and 150 mM NaCl, with the addition of 5 mM DTT and 1% sarkosyl to ensure maximum solubility of nucleic acids, effective inhibition of nuclease activity and removal of interfering contaminants (e.g. polysaccharides, polyphenols). The use of sodium acetate together with isopropanol shortened precipitation time and enhanced the yields of DNA/RNA. A phenol:chlorophorm:isoamyl alcohol step was subsequently used to purify the nucleic acids. The present protocol produces high yields of nucleic acids from only 25 mg of fresh algal biomass (0.195 and 0.284 µg mg^−1^ fresh weigh of RNA and DNA, respectively) and the high quality of the extracted nucleic acids was confirmed through spectrophotometric and electrophoretic analyses. The isolated RNA can be used directly in downstream applications such as RT-PCR and the genomic DNA was suitable for PCR, producing reliable restriction enzyme digestion patterns. Co-isolation of DNA/RNA from different strains indicates that this method is likely to have wider applications for intra- and inter-specific studies on other brown algae.

## Introduction

Brown algae are an ecologically and economically important group of marine photoautotrophs [1]–[4] that first appeared 200 million years ago and evolved multicellularity independently of green and red algae and higher plants [5], [6]. In 2007, the genome of *Ectocarpus siliculosus* (Dillwyn) Lyngbye, a filamentous brown alga of the order Ectocarpales, was published and it has been proposed as a model organism for brown algal genetic and genomic studies [7], [8], [9]. The species has certain characteristics such as a relatively small genome of 214 Mbp [8], a short life cycle that can be completed in laboratory culture [10], fast growth and ease of performing genetic crosses [7], [11], that makes it amenable to emerging molecular technologies.

At present, over 1500 strains of *E. siliculosus* have been isolated, from a broad range of geographic locations and ecological niches, and are maintained in culture collections [12]. Interestingly, intraspecific variations in copper tolerance [13], [14], as well as in the response to changes in salinity [15], [16], have been observed among strains of *E. siliculosus* isolated from different geographic locations; this variation is probably connected to a differential production of defence compounds or metabolites related to metal exclusion and metal chelation mechanisms, or in the accumulation of osmotically active compounds [14], [17]–[19].

This suggest the occurrence of genetic variability or plasticity, among the different strains of *E. siliculosus* and underlines that they provide a valuable resource for investigation of the molecular mechanisms underlying the dynamic responses of brown algae to abiotic and biotic stressors.

To perform molecular characterization a wide range of approaches are available (e.g. RT-PCR, qRT-PCR, microarray, cDNA library construction, SNP genotyping, DNA methylation profiling and next-generation sequencing), all requiring DNA and RNA samples of high purity [20]. The extracted nucleic acids need to be free of contaminants, including proteins, polysaccharides, polyphenols and lipids, but also of other nucleic acids; for example, it is important to obtain pure DNA-free RNA, suitable for sensitive downstream applications such as qRT-PCR, as well as DNA free of RNA that is a pre-requisite for performing downstream applications such as high throughput sequencing [21].

Besides quality, the integrity of the isolated nucleic acids will also directly affect the results of downstream applications [22]. Special precautions are required for RNA isolation as it has a very short half-life once extracted from cells or tissues and is susceptible to degradation [21], [23]–[25]. As for genomic DNA, each step of high throughput sequencing is exacerbated by degraded DNA that can result in loss of regions of the genome.

Currently, there are many specialized solution-based or column-based protocols for the extraction of pure DNA and RNA. Most of these protocols have been developed into commercial kits (e.g. TRIzol reagent, Invitrogen, Carlsbad, CA, USA or RNeasy kit, Qiagen, Valencia, CA, USA), that ease the extraction procedures. Although these protocols and commercial kits are commonly used for high quality nucleic acid extraction in model plants, they are unsuitable for organisms containing high levels of starch, polysaccharides and polyphenols [26]. Polysaccharides can co-absorb nucleic acids thus resulting in reduced yields and poor quality extracts, which, in the case of DNA, will interfere with endonuclease digestion [27]–[30]. Also, high concentrations of polyphenols, which can be co-extracted with nucleic acids and constitute strong enzyme inhibitors, can significantly impact the extraction procedure [31], [32].

Therefore, it is not surprising that for brown algae, which are particularly rich in problematic biomolecules, the isolation of pure nucleic acids represents a major challenge. In particular the isolation of nucleic acids is hindered by the presence of a chemically complex and dense cell wall [33]. Brown algal cell walls share some components with plants (cellulose) and animals (sulfated fucans), but they also contain some specific polysaccharides (alginates and laminarans) [34]–[36] that have structural, protective and storage roles [37]. Cellulose accounts for only a small proportion of the cell wall [38], with the main components being anionic polysaccharides [34]. Laminarans (or laminarins) comprise a mixture of linear β-(1,3)-glucans and branched -(1,6)-glucans (84–94% neutral sugar), with small amounts of uronic acid (6–9%) [35], [36], [39]. Alginates are linear copolymers of two uronic acids, β-1,4-D-mannuronate and α-1,4-L-guluronate residues, and fucoidans are sulfated polysaccharides containing α-L-fucose residues and a spectrum of highly ramified polysaccharides [34]–[36], [40]. In addition, brown algal cell walls contain phlorotannins [41], [42] and a small amount (∼5%) of proteins [43].

At present, several protocols are available for extracting nucleic acids from brown algae [27], [28], [44]–[48], including one for a specific strain of *E. siliculosus* (unialgal strain 32, CCAP accession 1310/4, origin san Juna de Marcona, Peru) [47]. However, due to intraspecific variation the concentrations of problematic biomolecules can vary between strains isolated from different geographic locations [13]–[19], consequently, it is necessary to develop a protocol that is strain/genotype-independent.

An additional problem is obtaining sufficient biomass for performing biochemical and molecular analyses. *Ectocarpus siliculosus* is a small filamentous alga that grows to a length of about 30 cm and does not yield large quantities of biological material during short-term experimental studies [11]. Existing protocols for obtaining good yields of DNA from *E. siliculosus* require 1 g of biomass [48]. Therefore, developing a protocol that relies on less biomass for nucleic acid extraction or the co-isolation of DNA and RNA from the same material would represent a significant breakthrough.

Thus, to address the issues of the purity of extracted nucleic acid, high nucleic acid yield from small quantities of biomass and strain-wide efficiency we have developed a rapid and effective method for the co-extraction of high-quality DNA and RNA starting from low biomass (25-, 50- and 100 mg) of *E. siliculosus*. To this end we have selected four strains (EC524, REP10–11, LIA4A, RHO12) originating from different locations in the southern and northern hemispheres and with different pollution histories and hence with differences in the concentrations of particular interfering metabolites [13]–[19]. A comparison between the protocol reported here and one previously used for *E. siliculosus* [47], highlights the significantly higher effectiveness of the new method.

Considering that *E. siliculosus* is the only model organism for brown algae and the phylogenetic distance of brown seaweeds from other photosynthetic organisms such as plants, red and green algae, we propose that the method presented here is a significant contribution to the field of research.

## Materials and Methods

Genomic DNA and total RNA were extracted from four randomly selected strains of *E. siliculosus.* The strains used originated from locations with different levels of metals pollution and have been maintained in control condition in the Plymouth University culture collection since 2010. They are: EC524 (from Chañaral, Chile a copper polluted site, (Accession number: 1310/333)); REP10–11, (from Restronguet Creek, England, a metal polluted site); LIA4A, (from Lon Liath, Scotland, a pristine site) and RHO12 (from Rhosneigr, Wales, a pristine site) (http://www.ccap.ac.uk/ccap_search.php?genus=Ectocarpus&strain=Ectocarpus%20siliculosus&mode=attr).

Collection of the seaweeds required no specific permission as sampling stations were not on privately-owned properties or from marine protected areas. This study did not involve endangered or protected species.

For nucleic acid extraction, strains were grown separately in 2 L polycarbonate bottles with standard culture medium, Provasoli Enriched Seawater (PES) [49] and the cultures were maintained in a controlled culture room (15°C (+/−1°C), 45 µmol photons m^−2 ^sec^−1^, 14/10 of light/dark cycle), and air bubbling to avoid CO_2_ depletion. Since the chemical composition of natural seawater can vary significantly between locations and seasons for experiments a synthetic, chemically defined, seawater medium, Aquil [50] was used. Prior to nucleic acid extraction, *E. siliculosus* was transferred from PES and acclimated in Aquil for 10 days.

Steps for the RNA-DNA co-isolation method will be described in the following 3 sections: Isolation of nucleic acids (Section 1.1), Purification (Section 1.2) and Quality control of nucleic acid (Section 1.3). A list of consumables, reagents, equipments and the guidelines of nucleic acids extraction are reported in the File S1.

### 1.1. Isolation of Nucleic Acids (Figure 1, Figure S1)

#### (a) Tissue harvesting

Different quantities of biomass (25-, 50- and 100 mg) of the four *E. siliculosus* strains were transferred into individual 2 mL microcentrifuge tubes, immediately frozen in liquid nitrogen and stored at −80°C to await extraction of the nucleic acids. To obtain the best quality of nucleic acids it is essential that harvested material is frozen rapidly and that the material is not allowed to thaw.

**Figure 1:**
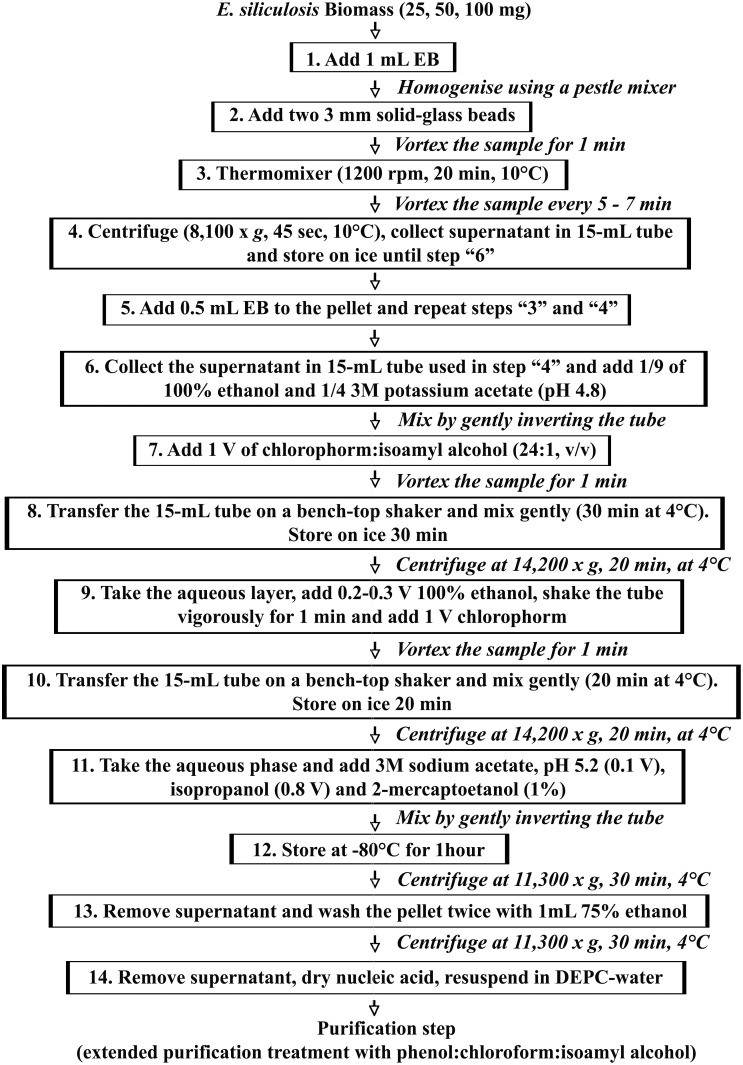
Summary of nucleic acids extraction from *E. siliculosus* brown alga. High yields of good quality DNA and RNA are isolated from as little as 25**Steps 1–5:** Harvested tissue is immediately homogenised using commercial 3 mm solid-glass beads in the presence of 1 mL EB containing 100 mM Tris-HCl, 150 mM NaCl, 5 mM DTT and 1% sarkosyl. These stages allow the lysis of the cell wall, the release of highest amount of nucleic acids, the inactivation of cellular nucleases, and the removal of most of the polysaccharides and other insoluble material. **Steps 6–10:** Simultaneous presence of absolute ethanol and potassium acetate aids polysaccharide precipitation. Moreover proteins, lipids, pigments and cell debris are removed through extraction of the aqueous phase with chloroform. **Steps 11–12:** Nucleic acids are then recovered by precipitation with 0.8 V of isopropanol and 0.1 V of 3 M sodium acetate (pH 5.2) in the presence of 1% 2-mercaptoetanol at −80°C. During the precipitation step, salts and other solutes are separated from nucleic acids that form a white precipitate collected by centrifugation. The excess of isopropanol and 2-mercaptoetanol are removed through washing the pellet with 75% ethanol. **Step 13:** All traces of ethanol are removed, the nucleic acid pellet is dried and resuspended in nuclease-free water. After RNase or DNase treatment the superfluous quantities of proteins, polysaccharides, lipids, and cell debris were removed from the extracted DNA and RNA through double extended purification treatment with phenol:chloroform:isoamyl alcohol.

#### (b) Cell lysis, inactivation of cellular nucleases and separation of nucleic acids from cell debris (Timing: 1 hour)

1. Prepare Extraction Buffer (EB: 100 mM Tris-HCl, pH 9.5; 150 mM NaCl; 1.0% sarkosyl). Add 5 mM DTT before use (Table S1). Once DTT is added the shelf-life of the buffer is only 2–3 days.
2. Add 1 mL of EB to each tube containing frozen algal material and with a blue pestle mixer, homogenise the tissue until the mixture thaws. Use a new pestle for each sample.
3. Add two 3 mm solid-glass beads to each tube and mix the contents vigorously, vortexing for 1 min. If processing multiple samples, leave the remaining samples on ice while carrying out steps 2 and 3.

**NOTE:** In this protocol, samples were not initially ground in liquid nitrogen to obtain a fine powder but were homogenised directly in EB as described in steps 2 and 3.

4. Transfer the samples to the thermomixer; mix and shake the samples at 1200 rpm for 20 min at 10°C. To aid effective tissue homogenisation, vortex samples every 5 min.
5. Centrifuge the samples for 45 sec at 8,100×*g* in an Eppendorf Minispin.
6. Collect the supernatant containing nucleic acids and transfer to a 15 mL tube. Keep on ice until step 9.
7. Repeat the extraction step by adding 0.5 mL EB to the 2 mL microcentrifuge tube, containing both pellet and glass beads. Shake vigorously for 1 min. Keep on ice if processing multiple samples.
8. Repeat steps 4 and 5.
9. Add supernatant to the 15 mL tube previously used in step 6 to obtain a final volume of 1.5 mL of extract.

#### (c) Removal of proteins and organic contaminants (Timing: 2–2.5 hours)

10. Add 1/9 volume of absolute ethanol (pre-cooled) and 1/4 volume of 3 M potassium acetate, (4.8 pH) (Table S2). Gently invert the tubes 8–10 times.

**NOTE:** The simultaneous presence of absolute ethanol and potassium acetate aids the precipitation of polysaccharides [51].

11. Add 2 mL of chloroform:isoamyl alcohol (24∶1, v/v) and shake the tube vigorously for 1 min. This step allows separation of nucleic acids from the mixture.

**NOTE:** The use of chloroform:isoamyl alcohol aids the removal of polysaccharides and proteins [26], [52].

12. Using a bench-top shaker, gently shake the 15 mL tube for 30 min at 4°C. Vortex the sample every 5–7 min during shaking. Incubate the tubes upright on ice for 30 min.
13. Centrifuge the sample at 14,200×*g* for 20 min at 4°C in order to separate the organic phase from the aqueous phase.
14. Carefully transfer the upper aqueous phase into a freshly prepared 15-mL tube placed on ice; add 0.2–0.3 volume of cold absolute ethanol and immediately shake the tube vigorously for 1 min. Vortex the tube immediately following addition of ethanol, to prevent nucleic acid precipitation.

**NOTE:** The addition of ethanol aids precipitation of polysaccharides [53].

15. Immediately add 2 mL (∼1 Volume) of chloroform and vortex vigorously for 1 min.
16. Using a benchtop shaker, mix the 15 mL tube for 20 min at 4°C. During the shaking, vortex samples every 5–7 min. Incubate the tubes upright on ice for 20 min.

#### (d) Precipitation of Nucleic acids (Timing: 2 hours)

17. Centrifuge samples at 14,200×*g* for 20 min at 4°C.
18. Distribute aliquots of the recovered aqueous phase into 2 mL conical tubes (Table 1, Figure S2).

**Table 1:** Split and precipitate the aqueous phase of one sample in more tubes (usually two).

e.g. SAMPLE 1	
1.5 mL aqueous phase	
750 µL aqueous phase	750 µL aqueous phase
(Sample 1A)	(Sample 1B)

19. Add the precipitation mix solution (0.8 V of isopropanol, 0.1 V of 3 M sodium acetate, (pH 5.2) and 1% of 2-mercaptoethanol) to each tube, in the order indicated in Table 2. Gently invert tubes 5–10 times.

**Table 2:** Reagent used in the precipitation step.

Aqueous Phase (top layer)		e.g. 1.5 mL		e.g. 1.4 mL	
Aqueous Phase split into two tubes		750 µL	750 µL	700 µL	700 µL
Precipitation mix	(0.8 V) Isopropanol	600 µL	600 µL	560 µL	560 µL
	(0.1 V) 3 M Sodium acetate,(pH 5.2)	75 µL	75 µL	70 µL	70 µL
	(1%) 2-mercaptoethanol	7.5 µL	7.5 µL	7 µL	7 µL

20. Precipitate the nucleic acids at −80°C for 1 h, or alternatively at −20°C overnight.
21. Centrifuge for 30 min at 11,300×*g* at 4°C to completely precipitate nucleic acids.
22. After centrifugation, discard the supernatant by inverting the tubes over a suitable container; if preferred, a pipette can be used to remove supernatant. Be careful not to dislodge the nucleic acid pellet.

#### (e) Washing DNA/RNA (Timing: 15 hours)

23. Wash the nucleic acid pellet twice with 1 mL of cold 75% ethanol to remove contaminants and any residual 2-mercaptoethanol; centrifuge at 11,300×*g* at 4°C for 20 min.
24. Remove any remaining traces of ethanol by pulse centrifugation and collect using a pipette (or by inverting the racked collection of tubes onto absorbent paper), and allow the pellet to air dry at room temperature under a laminar flow hood.

#### (f) Dissolving DNA/RNA (Timing: 20–40 minutes)

25. Hydrate the pellet with nuclease-free water (starting with 25–100 mg biomass the final volume should be between 40–50 µL); allow re-suspension on ice by gently shaking tubes. The samples can be stored at −20°C in the short-term but should be stored at −80°C for longer periods.

**NOTE:** In step 18, due to the high volume, the supernatant of one sample (e.g. Sample 1) was split and precipitated in two eppendorf tubes (e.g. Samples 1A and 1B) (Table 1, Figure S2). In this step it is possible to re-combine the nucleic acids from the two tubes (e.g. Samples 1A and 1B) into one tube (e.g. Sample 1) (Table 3, Figure S3).

**Table 3:** The nucleic acid of one sample precipitated in two different tubes is transferred in one tube after the resuspension in the appropriate volume of nuclease-free water.

25 µL resuspended nucleic acid	25 µL resuspended nucleic acid
(Sample 1A)	(Sample 1B)
50 µL resuspended nucleic acid	
(Sample 1)	

At this stage co-isolation of DNA and RNA was performed. To obtain pure DNA-free RNA, aliquots of nucleic acids should be treated with DNase enzyme in order to eliminate genomic DNA contamination.

Conversely, to obtain pure RNA-free DNA, aliquots of the nucleic acid mixture should be treated with RNase enzyme.

**NOTE:** By using primers that bridge exons, mixtures of nucleic acids can be used immediately for reverse transcription and qRT-PCR without DNase treatment [54]. Similarly, since RNA has a very short half-life once extracted, and does not impact DNA downstream processing, these can be performed without RNA digestion.

### 1.2. Purification Step

#### (a) RNase or DNase treatment

To obtain pure DNA, treat aliquots (10–25 µg) of nucleic acid mixtures with 1 µL of RNase A, DNase free enzyme (0.1 mg ml^−1^) (Roche Diagnostic Mannheim, Germany) in a final volume of 100 µL, for 20 min at 37°C.

To obtain pure RNA, treat aliquots (10–25 µg) of nucleic acid mixtures with 1 µL of DNase I recombinant, RNase free enzyme (10 U/µL) (Roche Diagnostic Mannheim, Germany) and 5 µL of 10X Incubation Buffer in a final volume of 50 µL and incubate for 17 min at 37°C.

#### (b) Purification of extracted DNA/RNA (Timing: 1 h)

When purifying nucleic acids it is important to use a method that maintains DNA/RNA integrity whilst removing contaminants. DNA or RNA was purified according the following procedure:

26. Add nuclease-free water to the nucleic acids (obtained at Step 1.2) to give a final volume of 500 µL.
27. Add 0.5 volume of phenol and vortex vigorously for 1 min.
28. Add 1 volume of chloroform:isoamyl alcohol (24∶1, v/v), vortex vigorously for 1 min.
29. Transfer the samples to the thermomixer, and shake the samples at a mixing speed of 1,300 rpm for 30 min at 10°C; vortex the samples every 5 min.
30. Centrifuge at 11,300×*g* for 25 min at 4°C;
31. Carefully collect the upper phase (avoiding mixing with the interphase layer) and repeat steps 27 to 30 if the interphase layer shows the presence of proteins/metabolites, identifiable by the presence of a white layer between the aqueous phase containing the nucleic acids and the organic phase containing the mixture of phenol:chloroform:isoamyl alcohol.
32. After centrifugation, carefully transfer the upper phase into freshly prepared 1.5 mL tubes. Add the precipitation mix solution (0.8 V of isopropanol, 0.1 V of 3 M sodium acetate, (pH 5.2) and 1% of 2-mercaptoethanol). Invert tubes to mix and incubate at −80°C for 1 h or at −20°C overnight.
33. Centrifuge samples at 11,300×*g* for 30 min at 4°C. Wash DNA/RNA pellets twice with 1 mL of cold 75% ethanol, dry and re-suspended in 40 µL of water.

### 1.3. Control of Nucleic Acid Quality

#### (a) Measuring DNA/RNA concentration and quality

Total DNA/RNA solutions, extracted from 25–100 mg of algae, were loaded on an agarose gel (1.5% w/v) for electrophoresis, stained with ethidium bromide (EtBr), and visualized under UV light to assess the quality and integrity of nucleic acids. Nucleic acid quantification was carried out by placing 1.5–2 µL in a Nanodrop spectrophotometer providing the absorbance ratios A_260_/A_280_ and A_260_/A_230_ that can be used to assess the presence of protein and polysaccharide/polyphenolic contamination [55]–[58].

**NOTE:** DNA/RNA concentrations and purity can also be determined spectrophotometrically by measuring absorbance at 230, 260 and 280 nm [52].

#### (b) Downstream applications of nucleic acids

Total RNA (1 µg) from each sample was reverse transcribed with the SuperScript III reverse transcriptase and oligo dT(22) according to the manufacturer’s instructions (Invitrogen, Milan). PCR and RT-PCR were performed to test DNA and cDNA quality, respectively. PCR was carried out in a 50-µL reaction mixture, which contained 70 ng template DNA or cDNA, 2.5 U Taq DNA polymerase (GoTaq, Promega), 1X Taq DNA polymerase buffer, 1.5 mM MgCl_2_, 0.2 µM each primer and 0.2 mM dNTPs.

Alpha Tubulin (TUA) was selected as a reference gene [47]. DNA amplification was done under the following conditions: 94°C for 2 min, followed by 40 cycles of 94°C for 50 s, 54°C for 50 s, and 72°C for 50 s, with a final extension at 72°C for 7 min. The PCR products (25 µl) were resolved on agarose gel (1% w/v) and visualized under UV light following EtBr staining.

#### (c) Downstream applications of nucleic acids: DNA digestion

Ten µg of extracted genomic DNA were restricted over night at 37°C with 60 U of 10 U/µl *Eco*RV enzyme (Fermentas, Milan, Italy), 20 µl of 10X *Eco*RV Buffer and 2 µl of 10 µg/µl BSA in a 200 µl final volume. The reaction was stopped by incubating at 65°C for 10 min. The digested DNA was precipitated at −20°C overnight in the presence of 0.1 V of 3 M sodium acetate (pH 5.2) and 2.5 V cold 100% ethanol. Samples were centrifuged at 11,300×*g* for 20 min at 4°C. The DNA pellet was washed with 1 mL of cold 75% ethanol, dried and re-suspended in 50 µl water. A 20 µl aliquot was rapidly checked by electrophoresis.

## Results

### 2.1. Yield of Genomic DNA and Total RNA

With the newly developed protocol, nucleic acid yields varied with initial quantity of biomass and between strains (Table 4, 5, S3, S4). For all strains, the absolute amount (µg) of purified nucleic acids extracted from 100 mg biomass was higher than that from 50- and 25-mg biomass. However, when quantities of nucleic acids were normalized to biomass (i.e. µg mg^−1^ of fresh weight) yield was highest from 25 mg biomass for three of the strains (RHO12; LIA4A; REP10–11) and from 50 mg for EC524 (Tables 4, 5). These results are consistent with a complete disintegration of tissue/cell structure in the extraction buffer when lower quantities of biomass (e.g. 25- and 50 mg) are used compared with the largest biomass (100 mg).

**Table 4:** Comparison of pure DNA yield and purity, obtained from four strains of *E. siliculosus* by two different methods: the new and old [47].

Strain		Starting Material Weight (mg fresh tissue)	A_260/280_		A_260/230_		DNA conc. (ng/µl)		Total DNA (µg)(a)		DNA Yield	
											(µg/mg)	
			New	Old	New	Old	New	Old	New	Old	New	Old
Polluted	REP 10.11	25	2.01±0.01	1.66±0.01	2.20±0.04	1.37±0.01	132.9±11.3	45.0±2.53	5.31±0.46	1.8±0.18	0.212±0.018*	0.071±0.006
		50	1.91±0.01	1.54±0.04	2.00±0.01	1.17±0.02	181.4±15.6	69.3±2.76	7.24±0.60	2.79±0.27	0.145±0.012*	0.056±0.009
		100	1.86±0.01	1.59±0.03	1.86±0.02	1.15±0.01	389.6±5.9	123.3±4.34	15.56±0.24	4.94±0.48	0.156±0.002*	0.049±0.006
	EC 524	25	1.96±0.03	1.59±0.01	1.75±0.02	1.36±0.01	96.5±5.6	58.0±2.51	3.86±0.42	2.32±0.45	0.155±0.015*	0.093±0.013
		50	1.92±0.01	1.60±0.02	1.66±0.03	1.27±0.01	213.4±10.6	124.3±5.43	8.54±0.55	4.98±0.84	0.171±0.017*	0.099±0.014
		100	1.85±0.02	1.56±0.02	1.65±0.03	1.15±0.01	314.6±5.7	139.5±4.82	12.56±0.75	5.57±0.62	0.126±0.016*	0.056±0.006
Pristine	LIA 4A	25	1.91±0.01	1.25±0.02	1.76±0.02	1.61±0.01	274.7±16.6	226.2±6.92	10.97±0.42	9.04±0.58	0.438±0.029*	0.36±0.009
		50	1.87±0.01	1.19±0.01	1.73±0.02	1.62±0.02	357.1±7.5	332.0±7.43	14.26±0.96	13.26±1.34	0.284±0.024	0.26±0.007
		100	1.81±0.02	1.20±0.02	1.73±0.02	1.59±0.02	653.9±40.8	515.4±5.73	26.14±1.28	20.62±2.65	0.261±0.031*	0.21±0.008
	RHO 12	25	1.83±0.02	1.25±0.03	1.63±0.01	0.69±0.02	207.6±2.62	93.5±3.23	8.30±0.11	3.74±0.83	0.332±0.004*	0.15±0.009
		50	1.80±0.01	1.19±0.02	1.60±0.01	0.67±0.02	307.2±15.2	157.9±2.11	12.28±0.61	6.33±0.95	0.246±0.012*	0.13±0.01
		100	1.80±0.02	1.15±0.03	1.61±0.01	0.63±0.01	390.6±52.1	253.0±2.43	15.60±2.08	10.10±2.41	0.156±0.020	0.10±0.01

Total amounts of nucleic acids were calculated in a final volume of 40 µL.

(a) Data are reported as means ± SE from five independent nucleic acid extractions, for both methods. ‘New’ refers to the method developed in this study; ‘Old’ refers to a previously published protocol based on CTAB extraction buffer. According to one-way ANOVA and post-hoc Tukey Test at 95% confidence interval, an asterisk (*) indicates the significantly differences between the yields of the two methods.

**Table 5:** Comparison of pure RNA yield and purity, obtained from four strains of *E. siliculosus* by two different methods: the new and old [47].

Strain		StartingMaterialWeight(mg fresh tissue)	A_260/280_		A_260/230_		RNA conc. (ng/µl)		Total RNA (µg)(a)		RNA Yield	
											(µg/mg)	
			New	Old	New	Old	New	Old	New	Old	New	Old
Polluted	REP 10.11	25	2.01±0.01	1.68±0.01	2.40±0.08	1.36±0.02	110.9±5.86	33.2±1.05	4.43±0.23	1.33±0.04	0.176±0.009*	0.053±0.002
		50	1.90±0.01	1.51±0.01	2.00±0.01	1.18±0.02	153.6±4.74	60.3±1.59	6.14±0.19	2.41±0.06	0.124±0.004*	0.048±0.001
		100	1.84±0.02	1.64±0.02	1.85±0.02	1.11±0.02	295.4±13.2	113.3±1.56	11.82±0.52	4.54±0.05	0.118±0.005*	0.045±0.0005
	EC 524	25	1.96±0.03	1.64±0.01	1.75±0.01	1.34±0.01	66.5±2.68	44.2±2.03	2.66±0.11	1.77±0.07	0.106±0.005*	0.071±0.003
		50	1.90±0.02	1.62±0.01	1.63±0.01	1.25±0.01	184.8±15.8	110.3±1.37	7.30±0.63	4.42±0.06	0.144±0.012*	0.088±0.001
		100	1.84±0.01	1.52±0.01	1.62±0.02	1.12±0.01	271.0±2.20	129.0±1.62	10.82±0.07	5.16±0.07	0.108±0.0007*	0.052±0.001
Pristine	LIA 4A	25	1.89±0.02	1.25±0.04	1.73±0.01	1.62±0.01	155.6±5.45	124.2±1.23	6.20±0.22	4.96±0.04	0.250±0.009	0.2±0.002
		50	1.87±0.02	1.16±0.02	1.71±0.01	1.60±0.02	233.5±4.91	227.0±3.26	9.34±0.20	9.10±0.13	0.186±0.002	0.18±0.003
		100	1.79±0.01	1.20±0.01	1.71±0.02	1.58±0.01	514.7±3.32	475.4±0.02	20.56±0.15	19.0±0.24	0.205±0.001	0.19±0.002
	RHO 12	25	1.84±0.02	1.24±0.03	1.63±0.01	1.08±0.01	154.2±13.8	88.3±1.30	6.17±0.55	3.54±0.05	0.244±0.022*	0.14±0.002
		50	1.83±0.01	1.17±0.01	1.60±0.01	1.09±0.02	174.7±8.9	131.0±1.12	6.98±0.36	5.22±0.04	0.140±0.007*	0.10±0.0008
		100	1.86±0.03	1.16±0.01	1.62±0.02	1.06±0.01	311.6±20.1	249.0±1.38	12.46±0.79	9.95±0.05	0.125±0.008*	0.10±0.0005

Total amounts of nucleic acids were calculated in a final volume of 40 µL.

(a) Data are reported as means ± SE from five independent nucleic acid extractions, for both methods. ‘New’ refers to the method developed in this study; ‘Old’ refers to a previously published protocol based on CTAB extraction buffer. According to one-way ANOVA and post-hoc Tukey Test at 95% confidence interval, an asterisk (*) indicates the significantly differences between the yields of the two methods.

As a general rule, higher yields of both DNA and RNA were obtained from the selected strains (RHO12; LIA4A; REP10-11; EC524) using the new protocol than the CTAB extraction buffer method [47] (Tables 4, 5, S3, S4, S5).

### 2.2. Purity of Genomic DNA and Total RNA

The quality of nucleic acids obtained for all four strains (RHO12; LIA4A; REP10–11; EC524), was better than that of the CTAB extraction buffer method [47].

The purity of nucleic acids depended on both the quantity of initial biomass and the strain. In general, 25 mg biomass provided the highest level of purity and when used in the co-isolation, the A_260_/A_280_ ratios ranged between 1.8 and 2.0, whilst the A_260_/A_230_ ratios ranged between 1.6 and 2.4 (Tables 4, 5, S3, S4). These values indicate that the DNA and RNA samples were effectively separated from both proteins and polysaccharides (Tables 4, 5).

For REP10–11, the respective ratios ranged between 1.8 and 2.0, and 1.8 and 2.4, respectively, and were independent of the quantity of biomass used (Tables 4, 5, Figure S4). Regardless of the amount of biomass or strain used, the nucleic acids extracted through this protocol were successfully used for downstream applications.

### 2.3. Quality and Integrity of Genomic DNA and Total RNA

**Figure 2:**
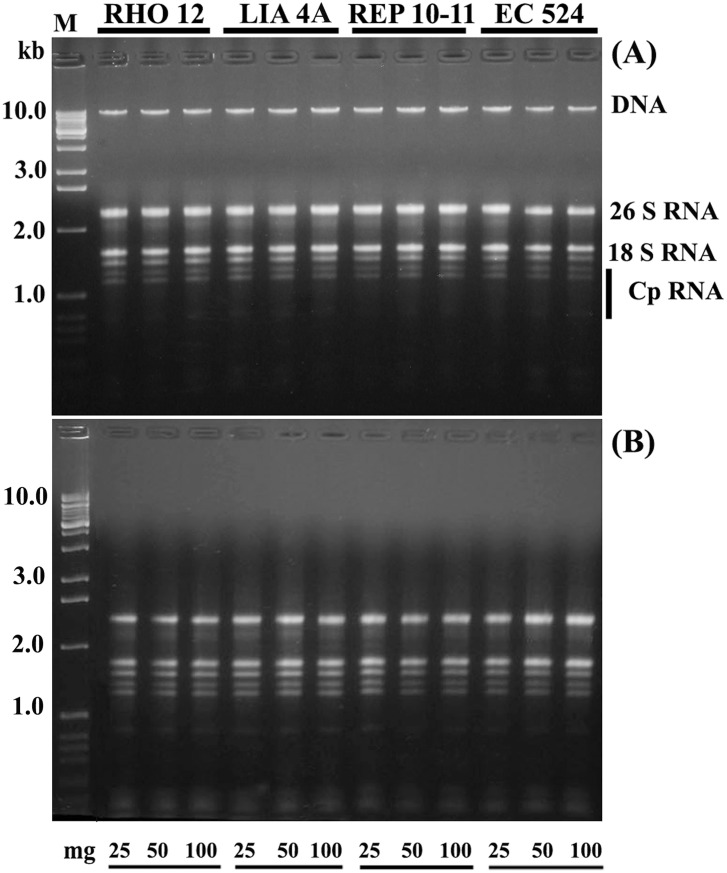
Analysis of quality and integrity of extracted nucleic acids. (**A**) Genomic DNA and total RNA (∼0.5 µg) isolated simultaneously from four strains of *E. siliculosus* (RHO12; LIA4A; REP10–11; EC524), using initial biomass of 25, 50 and 100 mg (gel stained with ethidium bromide). DNA shows an intact single band whilst RNA shows the clear cytosolic and plastid (Cp) ribosomal bands. (**B**) Genomic DNA contamination is effectively removed by DNase treatment, whilst the pure RNA retains intactness and quality. RNA species of low molecular weight are also apparent. M: RNA Ladder, High Range (Fermentas, Italy).

The integrity of nucleic acids was examined by 1.5% (w/v) agarose gel electrophoresis (Figure 2). For co-isolated nucleic acids (Figure 2A), a distinct individual band of DNA and cytosolic and plastid ribosomal RNA bands were observed. After the purification steps, RNA intactness and the absence of DNA contamination was evident from the electrophoretic pattern that shows only cytosolic and plastid ribosomal RNA bands (Figure 2B). Similarly, the absence of DNA degradation is evidenced by an electrophoretic pattern showing only a distinct individual band of DNA (Figure 2A, 3A). These results confirm that highly purified nucleic acids were obtained, which can be used in downstream applications.

**Figure 3:**
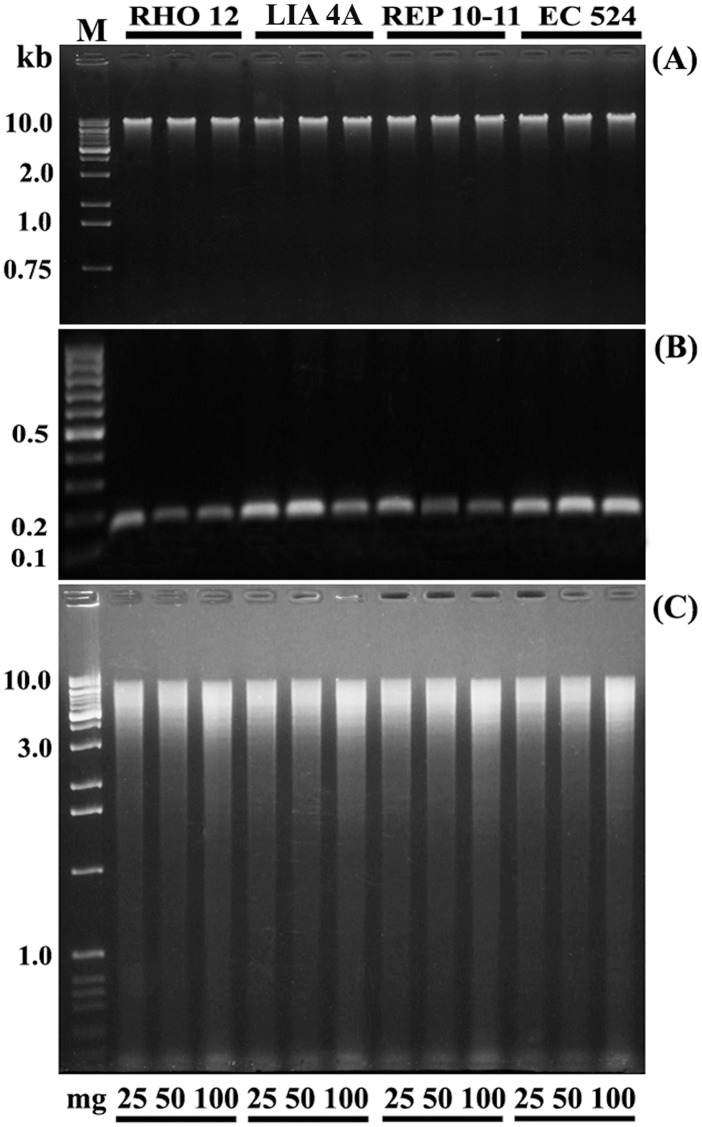
Gel electrophoresis analysis of pure DNA and its downstream application. (**A**) Genomic DNA (∼0.5 µg), after RNase treatment, isolated from strains of *E. siliculosus* (RHO12, LIA4A; REP10–11; EC524) using initial biomass of 25, 50 and 100 mg. (**B**) The quality of isolated DNA was confirmed by electrophoresis analysis of a DNA PCR product using an alpha tubuline (TUA) housekeeping gene. (**C**) Electrophoretic analysis of *Eco*RV enzyme digestion product of genomic DNA confirms that the extracted DNA is suitable for downstream application (gels stained with ethidium bromide). M: 100-bp, 1-Kb DNA and High Range RNA Ladder (Fermentas, Italy).

### 2.4. Downstream Applications

**Figure 4:**
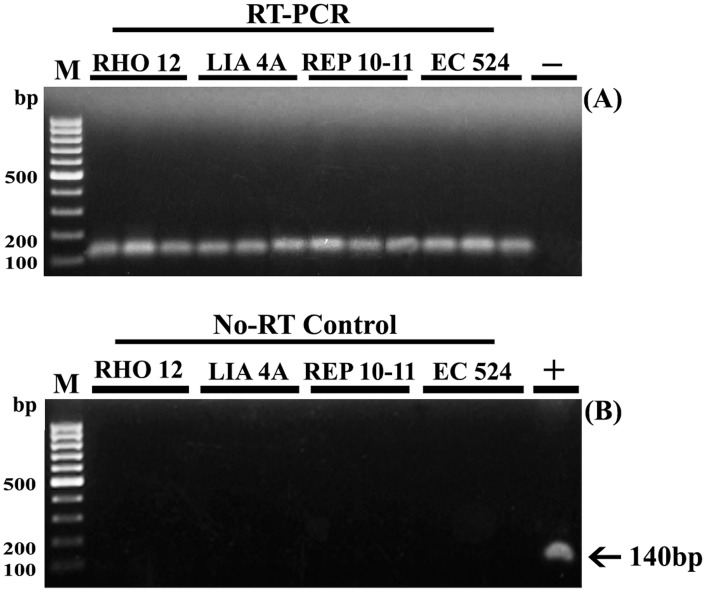
RT-PCR analysis of TUA expression of four strains of *E. siliculosus.* RNA samples extracted from four strains of *E. siliculosus* (RHO12, LIA4A; REP10–11; EC524) using initial biomass of 25, 50 and 100 mg were analyzed by RT-PCR for the alpha tubuline (TUA) housekeeping gene. No amplification was observed when RNA was directly used for PCR (No-RT control panel), indicating that no DNA contamination is present in the RNA starting material. M: 100-bp DNA ladder (Fermentas, Italy); −: PCR negative control (no DNA, but water was added); +: PCR positive control (no RNA but DNA was added).

The quality of the extracted genomic DNA was further confirmed by results of PCR amplification and enzyme digestion performed using DNA from all strains and initial quantities of biomass (Figure 3B, 3C, Figure S5). In all cases, agarose gel analysis revealed that a 140-bp of the Alpha Tubulin (TUA) housekeeping gene was amplified (Figure 3B), and the extracted genomic DNA was successfully digested by *Eco*RV restriction enzyme (Figure 3C, Figure S5). Similarly, the intactness and quality of the obtained total RNA for downstream applications was tested through RT-PCR analysis (Figure 4). The total RNA obtained from all strains was sufficiently pure for the successful conversion into cDNA, regardless of the amount of biomass used. Moreover, the cDNA obtained was successfully used in the amplification process, by using a specific Alpha Tubulin primer pair (Figure 4). This result confirms that the total RNA was of high integrity and the mRNA was intact.

## Discussion

To the best of our knowledge, the protocol outlined here is the first to allow the co-isolation of highly pure genomic DNA and intact RNA from different strains of *Ectocarpus siliculosus* using small quantities of biomass.

Obtaining high-quality nucleic acids is the primary and most critical step in molecular biology studies, particularly when using difficult material such as brown algae. The presence of cell walls composed of cellulose, sulfated fucans, laminarans and alginates [33]–[37], [41]–[43] together with high concentrations of metabolites such as lipids and polyphenols that can cross-link and contaminate nucleic acids have hindered the development of an effective low-cost and time-efficient extraction protocol for brown algae.

The protocol reported in this paper is rapid, relatively non-toxic, inexpensive, and applicable for extracting large quantities of high purity DNA and RNA from small amounts (down to 25 mg) of biomass of *E. siliculosus* strains isolated from different environmental conditions.

The critical steps of the presented protocol include cell lysis, to destroy the cellular structure (cell walls and membranes), inactivation of cellular nucleases, separation of desired nucleic acids from cell debris and contaminants and purification of DNA and RNA. Due to the dense and complex nature of brown algal cell walls [33]–[37], [41]–[43], in this new protocol we selected a detergent-based cell lysis in conjunction with homogenization and mechanical grinding to effectively lyse cells. The mechanical method employs very small (3 mm) glass beads which in the extraction buffer (EB) disrupts the sample through high level agitation by shaking. This approach has been successfully applied for nucleic acid extraction from difficult plant tissues [59]. Its advantages over other methods (e.g. grinding tissue with liquid nitrogen using a mortar or use a probe sonicator), are in the ability to process many samples at a time with no concerns of cross-contamination, and to disrupt very small samples and hence use low biomass which is an important consideration when working with *E. siliculosus*.

Lysis of cells leads to the release of large quantities of contaminants that can impede DNA and RNA extraction and/or inhibit analytical studies on the isolated nucleic acids [60]. Therefore, we developed an EB (pH 9.5, containing 100 mM Tris-HCl, 150 mM NaCl, 5 mM DTT and 1% sarkosyl) that, not only destroyed cells, but ensured maximum solubility of nucleic acids, resulting in effective inhibition of RNase/DNase activity and in the removal of interfering insoluble material.

Strong detergents such as SDS (sodium dodecyl sulfate) and sarkosyl (N-lauroyl sarcosine or sarcosine) have been used to extract nucleic acids from mammals [61], [62], plants [63], [64] and seaweeds [65], [66] by inducing membrane dissociation, solubilization and precipitation of membrane lipids, protein denaturation, and dispersion of protein aggregates [67]–[69]. In our method, and in agreement with previously reported data [70], 1% sarkosyl and 150 mM NaCl proved to be effective in removing most of the proteins, polyphenols and polysaccharides, and in releasing the highest quantities of nucleic acids.

The inclusion of dithiothreitol (5 mM DTT) in the EB is another critical component of our protocol. Compared to the most commonly used anti-oxidant, β-mercaptoethanol, DTT has a stronger reducing capacity that prevents oxidative cross-linking of nucleic acids by phenolics, and inhibition of nucleases activity by disrupting disulphide bond formation [71].

Potassium acetate was then used to further reduce the concentrations of polysaccharides, which are precipitated as potassium salts; this approach has been widely used for RNA extraction from plants [51], [53], [72]–[74]. Subsequent extraction by chloroform-isoamyl alcohol led to a compact inter-phase compound that makes the transfer of aqueous phase, which contains the nucleic acids, a much easier task. The slow addition of absolute ethanol into the recovered aqueous phase, followed by a second chloroform extraction, allows the nucleic acids to remain in solution, while polysaccharides form a jelly-like precipitate [51], [53], [75]. Chloroform is also used during nucleic acids extraction, due to its ability to denature proteins, thereby dissociating nucleic acids from them [26], [52]. In addition to removal of polysaccharides and proteins this treatment also aids in eliminating different pigments, such as chlorophylls and fucoxanthin, one of the most abundant carotenoids of brown algae [76].

To date, different methods have been used to remove polysaccharide and phenolic contamination from nucleic acids extracted from plants [64], [77], [78]. EBs containing high salt concentrations, such as NaCl (1.0–2.5 M) have been commonly used in the extraction of starch-rich tissues [53], [79], [80], but its presence can result in a significant reduction in RNA yield when isolated from polysaccharides-rich tissues [70]. Standard RNA extraction methods using guanidine isothiocyanate-phenol-chloroform [26], or RNeasy kits have failed to provide satisfactory yield and purity of RNA when attempting to extract it from starch-rich tissues. Moreover, CTAB, widely used to remove contaminating polysaccharides [81], [82], has not provided DNA amenable to enzyme-restriction digestion when applied to green algae [83]. In agreement with this latter research, the yields and purity of DNA and RNA from *E. siliculosus* samples (RHO12; LIA4A; REP10-11; EC524) were very low when we used the CTAB extraction method [47].

Proteinase K is often used to separate proteins from nucleic acids and inhibit ribonucleases [48], [84], [85]. However, in many of the protocols used for extracting nucleic acids from brown algae this component is lacking [27], [44], [46], [47], [65], [86]. In addition to potential issues related to the temperature of proteinase K action (∼37–56°C), the strong activity of this enzyme makes it difficult to optimize conditions for proteolytic digestion [52], especially when applied to different strains, as was the case with *E. siliculosus*.

After RNase or DNase treatment, the extracted DNA or RNA was further purified through double extended treatment with phenol:chloroform:isoamyl alcohol [26], [52], [87]. As a consequence of this treatment a polar aqueous phase, containing DNA or RNA was separated from a non-polar organic phase, which contained the contaminants. Nucleic acids in the supernatant were precipitated using isopropanol and 3 M sodium acetate (pH 5.2) in the presence of 2-mercaptoethanol at −80°C [46], [88]. During nucleic acids precipitation, salts and other solutes, such as residual phenol and chloroform, remain in solution while nucleic acids form a white precipitate that can be easily collected by centrifugation.

Using the described method, high yields of integral and pure genomic DNA and total RNA were extracted, as confirmed by spectrophotometric and electrophoretic analyses. The purity of nucleic acids from protein contamination is commonly measured by calculating the ratio A_260_/A_280_, while the level of organic contaminants, e.g. polysaccharides and polyphenols, is determined from the ratio A_260_/A_230_ [55]–[58]. The values we obtained indicate that both DNA and RNA samples were pure and effectively separated from protein, polysaccharides and other metabolites, and that the quality of the extracted nucleic acids was strongly improved compared with the CTAB extraction method [47]. In general, for all four strains used, the highest level of purity was obtained from 25 mg, followed by 50 and 100 mg biomass.

The highest yields of total DNA and RNA (0.284 and 0.195 µg mg^−1^ fresh weight respectively) were also obtained from a biomass of 25 mg. This result is highly significant as in previous studies on *Fucus vesiculosus* and *Saccharina japonica,* comparable yields of extracted nucleic acids required 250 and 500 mg of biomass, respectively [44], [46]. Therefore, we strongly recommend using small quantities of starting material for extracting nucleic acids from brown algae.

The integrity of the nucleic acid samples was examined on a 1.5% agarose gel. All RNA samples were intact as judged by the sharp and distinct cytosolic and plastid ribosomal bands on the agarose gel. Moreover, agarose gel electrophoresis showed a distinct individual band of intact genomic DNA as well as reliable restriction enzyme digestion patterns. The absence of smear on the gel confirms the spectrophotometric results, and provides further evidence that this protocol efficiently removed contaminants during DNA and RNA isolation from the different strains of *E. siliculosus*.

Consistent with the high quality of nucleic acids obtained through this method the RNA was suitable for RT-PCR, allowing its efficient use in sensitive downstream applications such as qRT-PCR assays and next-generation technologies. Similarly, the genomic DNA, free of interfering compounds, was efficiently used for PCR and therefore would be suitable for DNA sequencing, southern blot hybridization and whole genome methylation sequencing. Interestingly, a method [47] previously used to isolate RNA from a specific strain of *E. siliculosus* (strain Es32, CCAP accession 1310/4, originating from San Juna de Marcona, Peru) did not produce the same levels of yield and purity when applied to the four strains used in this study. Furthermore, although the effectiveness of the recently published protocol by Coelho et al. [48] for isolating genomic DNA from *E. siliculosus* (strain not specified) was not assessed in this study, the quantity of biomass required (1 g F.W.) far exceeded the amount used in the method reported here.

In conclusion, we have developed a protocol for the co-isolation of high-quality DNA and RNA from the model brown alga *E. siliculosus,* that should expedite studies aimed at understanding biological functions of brown seaweeds, an ecologically and economically important group of coastal and estuarine photoautotrophs from cold and temperate latitudes. Despite the problematic metabolites present in the cell and associated with the cell wall, the DNA and RNA extracted were of excellent quality and applicable for downstream applications. Together with the spectrophotometric and electrophoretic analyses these results provide evidence that the method successfully dealt with these interfering components. Moreover, by using this protocol it is possible to obtain high yields of nucleic acids from small quantities of biomass, and both yield and purity are strain-independent. We further suggest that the protocol may have wider applicability to other algal species that have polyphenol- and polysaccharide-rich tissues.

## Supporting Information

Figure S1 **Summary of nucleic acids extraction from** ***Ectocarpus siliculosus.***
(PPT)

Figure S2 **Nucleic acids precipitation.** At this step it is possible to precipitate the nucleic acids by splitting the aqueous phase of one sample in multiple tubes (usually two), and in a second step join the precipitated nucleic acids.
(DOC)

Figure S3 **The nucleic acids of one sample are combined in single tube.** After resuspension in an appropriate volume of nuclease-free water, the nucleic acids precipitated in two different tubes (step 18) should be transferred into a new tube, to obtain a final volume of 40–50 µL.
(DOC)

Figure S4 **Nanodrop spectrophotometry measurements of REP10.11 extracted RNA.** Total RNA extracted from REP10–11, measured after DNase treatment and a purification step, are of high quality and free from appreciable levels of organic contaminants regardless of the biomass used in the extraction procedures. (A) 25 mg (B) 50 mg and (C) 100 mg of starting biomass, respectively.
(TIF)

Figure S5 **Comparison of undigested and** ***Eco*** **RV digested DNA.** Genomic DNA (10 µg) of *E. siliculosus* strains (RHO12, LIA4A, REP10–11, EC524 from 25, 50 and 100 mg biomass) was digested with *Eco*RV enzyme (60 units in 200 µl at 37°C, over night) followed by electroforesis on 0.8% agarose gel. The undigested DNA was incubated under the same conditions but without *Eco*RV enzyme. M: 100 bp ladder.
(TIF)

Table S1 **Extraction Buffer (EB) guideline.**
(DOC)

Table S2 **Reagent used to remove contaminants.**
(DOC)

Table S3 **Comparisons of mean values of pure DNA yield and purity between strains isolated from polluted sites (REP10.11, EC524) and those from pristine sites (LIA4A, RHO12).** Strains collected from pristine sites exhibit a higher quantity of nucleic acids extracted compared to those from polluted sites. Total amounts of nucleic acids (µg) were calculated in a final volume of 40 µL (a). Data are reported as means ± SE from five independent nucleic acid extractions. Different letters in the DNA yield column represent significant differences according to one-way ANOVA and post-hoc Tukey Test at 95% confidence interval.
(DOC)

Table S4 **Comparisons of mean values of pure RNA yield and purity between strains isolated from polluted sites (REP10.11, EC524) and those from pristine sites (LIA4A, RHO12).** Strains collected from pristine sites exhibit a higher quantity of nucleic acids extracted compared to those from polluted sites. Total amounts of nucleic acids (µg) were calculated in a final volume of 40 µL (a). Data are reported as means ± SE from five independent nucleic acid extractions. Different letters in the RNA yield column represent significant differences according to one-way ANOVA and post-hoc Tukey Test at 95% confidence interval.
(DOC)

Table S5 **Mean nucleic acids yield reduction (%) obtained with the old method.** A differential decrease in the quantity of nucleic acids was recorded for all strains when the old method [47] was used compared with the new one.
(DOC)

File S1 **List of consumables, solutions and reagents, equipment as well as a guideline of nucleic acids extraction.**
(DOC)
