# Correction: A Simple and Effective Method for High Quality Co-Extraction of Genomic DNA and Total RNA from Low Biomass *Ectocarpus siliculosus*, the Model Brown Alga

**DOI:** 10.1371/journal.pone.0101190

**Published:** 2014-07-08

**Authors:** The PLOS ONE Staff

There are errors in Tables 1-3. The author has provided corrected tables below. The publisher apologizes for these errors.

**Table 1:** Split and precipitate the aqueous phase of one sample in more tubes (usually two).

	e.g. SAMPLE 1	
	1.5 mL aqueous phase	
**750 µL aqueous phase**		**750 µL aqueous phase**
**(Sample 1A)**		**(Sample 1B)**

**Table 2:** Reagent used in the precipitation step.

Aqueous Phase (top layer)			e.g. 1.5 mL		e.g. 1.4 mL	
Aqueous Phase split into two tubes			750 μL	750 μL	700 μL	700 μL
**Precipitation mix **		(0.8 V) Isopropanol	600 μL	600 μL	560 μL	560 μL
		(0.1 V) 3 M Sodium acetate, (pH 5.2)	75 μL	75 μL	70 μL	70 μL
		(1%) 2-mercaptoethanol	7.5 μL	7.5 μL	7 μL	7 μL

**Table 3:** The nucleic acid of one sample precipitated in two different tubes is transferred in one tube after the resuspension in the appropriate volume of nuclease-free water.

25 µL resuspended nucleic acid					25 µL resuspended nucleic acid				
	**(Sample 1A)**						**(Sample 1B)**		
		**50 µL resuspended nucleic acid**							
				**(Sample 1)**					
