# Supplementary material for: A Simple and Effective Method for High Quality Co-Extraction of Genomic DNA and Total RNA from Low Biomass Ectocarpus siliculosus, the Model Brown Alga
Source: PLoS One. 2014 May 27;9(5):e96470. doi: 10.1371/journal.pone.0096470 (PMC4035266; doi:10.1371/journal.pone.0096470)
Supplement: Figure S1 — Summary of nucleic acids extraction from Ectocarpus siliculosus. (PPT) [file pone.0096470.s001.ppt]

## Slide 1
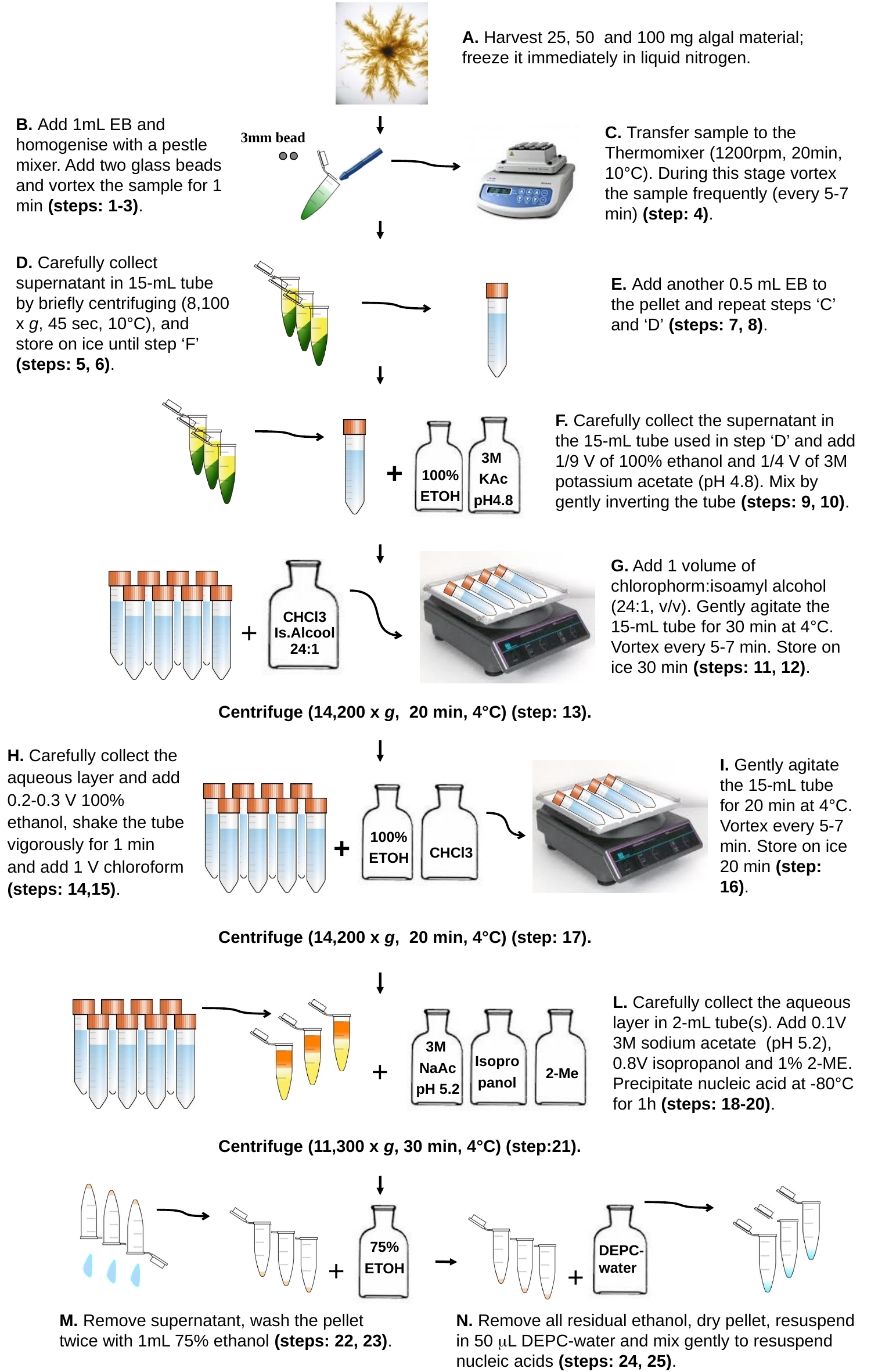

A. Harvest 25, 50 and 100 mg algal material; freeze it immediately in liquid nitrogen.
B. Add 1mL EB and homogenise with a pestle mixer. Add two glass beads and vortex the sample for 1 min (steps: 1-3).
C. Transfer sample to the Thermomixer (1200rpm, 20min, 10°C). During this stage vortex the sample frequently (every 5-7 min) (step: 4).
3mm bead
D. Carefully collect supernatant in 15-mL tube by briefly centrifuging (8,100 x g, 45 sec, 10°C), and store on ice until step ‘F’ (steps: 5, 6).
E. Add another 0.5 mL EB to the pellet and repeat steps ‘C’ and ‘D’ (steps: 7, 8).
F. Carefully collect the supernatant in the 15-mL tube used in step ‘D’ and add 1/9 V of 100% ethanol and 1/4 V of 3M potassium acetate (pH 4.8). Mix by gently inverting the tube (steps: 9, 10).
3M
KAc
pH4.8
+
100%
ETOH
G. Add 1 volume of chlorophorm:isoamyl alcohol (24:1, v/v). Gently agitate the 15-mL tube for 30 min at 4°C. Vortex every 5-7 min. Store on ice 30 min (steps: 11, 12).
+
CHCl3
Is.Alcool
24:1
Centrifuge (14,200 x g, 20 min, 4°C) (step: 13).
H. Carefully collect the aqueous layer and add 0.2-0.3 V 100% ethanol, shake the tube vigorously for 1 min and add 1 V chloroform (steps: 14,15).
I. Gently agitate the 15-mL tube for 20 min at 4°C. Vortex every 5-7 min. Store on ice 20 min (step: 16).
+
100%
ETOH
CHCl3
Centrifuge (14,200 x g, 20 min, 4°C) (step: 17).
L. Carefully collect the aqueous layer in 2-mL tube(s). Add 0.1V 3M sodium acetate (pH 5.2), 0.8V isopropanol and 1% 2-ME. Precipitate nucleic acid at -80°C for 1h (steps: 18-20).
3M
NaAc
pH 5.2
+
Isopro
panol
2-Me
Centrifuge (11,300 x g, 30 min, 4°C) (step:21).
75%
ETOH
DEPC-water
+
+
M. Remove supernatant, wash the pellet twice with 1mL 75% ethanol (steps: 22, 23).
N. Remove all residual ethanol, dry pellet, resuspend in 50 μL DEPC-water and mix gently to resuspend nucleic acids (steps: 24, 25).
