# Supplementary material for: A Simple and Effective Method for High Quality Co-Extraction of Genomic DNA and Total RNA from Low Biomass Ectocarpus siliculosus, the Model Brown Alga
Source: PLoS One. 2014 May 27;9(5):e96470. doi: 10.1371/journal.pone.0096470 (PMC4035266; doi:10.1371/journal.pone.0096470)
Supplement: Figure S2 — Nucleic acids precipitation. At this step it is possible to precipitate the nucleic acids by splitting the aqueous phase of one sample in multiple tubes (usually two), and in a second step join the precipitated nucleic acids. (DOC) [file pone.0096470.s002.doc]

**Greco et al., Figure S2.**
