# Supplementary figures and images for: A Simple and Effective Method for High Quality Co-Extraction of Genomic DNA and Total RNA from Low Biomass Ectocarpus siliculosus, the Model Brown Alga
Source: PLoS One. 2014 May 27;9(5):e96470. doi: 10.1371/journal.pone.0096470 (PMC4035266; doi:10.1371/journal.pone.0096470)

**
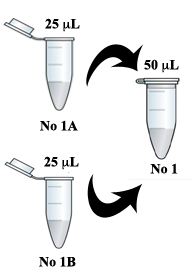
**

**Greco et al., Figure S3.**

Supplement: Figure S3 — The nucleic acids of one sample are combined in single tube. After resuspension in an appropriate volume of nuclease-free water, the nucleic acids precipitated in two different tubes (step 18) should be transferred into a new tube, to obtain a final volume of 40–50 µL. (DOC) [file pone.0096470.s003.doc]

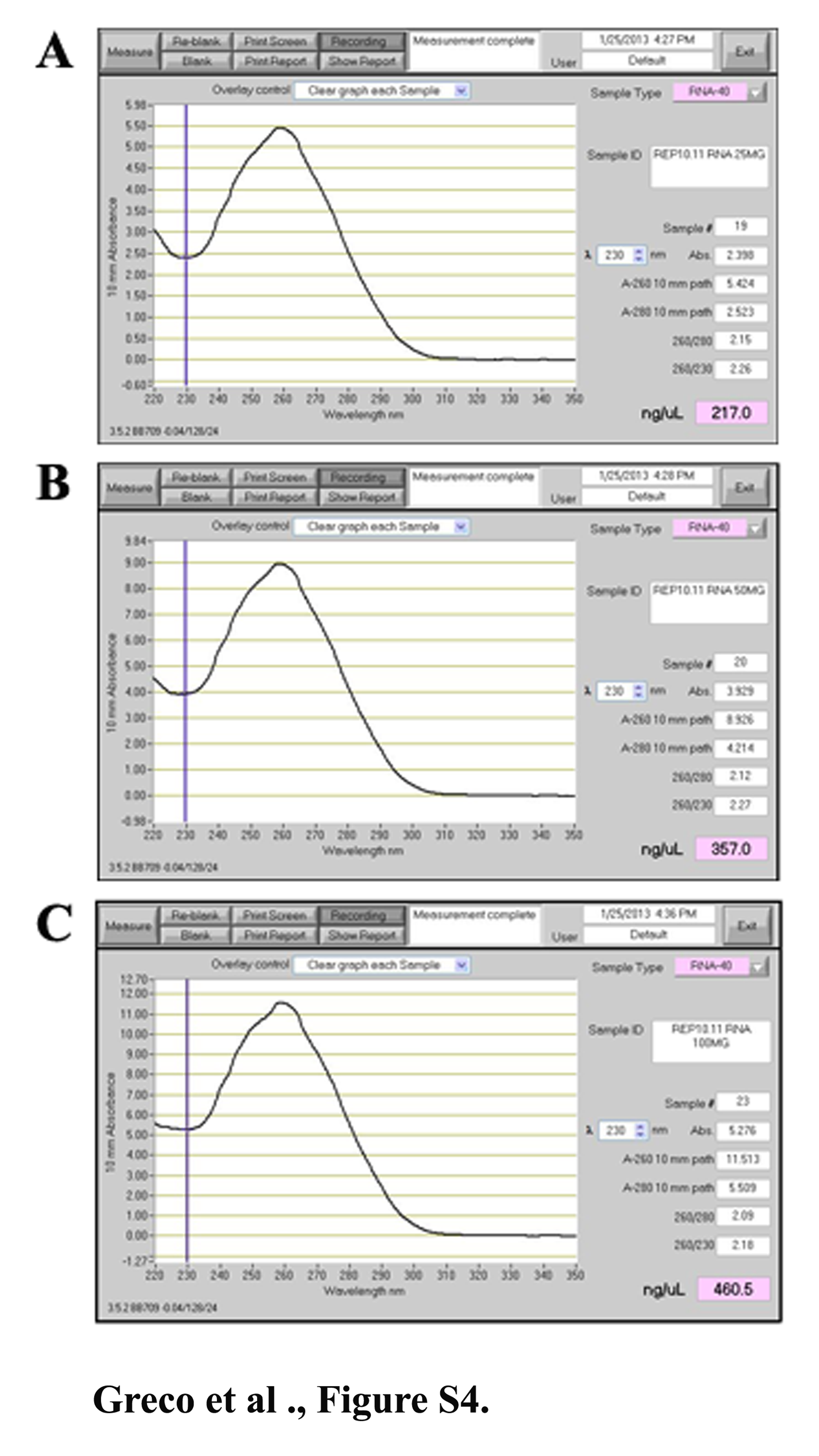

Supplement: Figure S4 — Nanodrop spectrophotometry measurements of REP10.11 extracted RNA. Total RNA extracted from REP10–11, measured after DNase treatment and a purification step, are of high quality and free from appreciable levels of organic contaminants regardless of the biomass used in the extraction procedures. (A) 25 mg (B) 50 mg and (C) 100 mg of starting biomass, respectively. (TIF) [file pone.0096470.s004.tif]

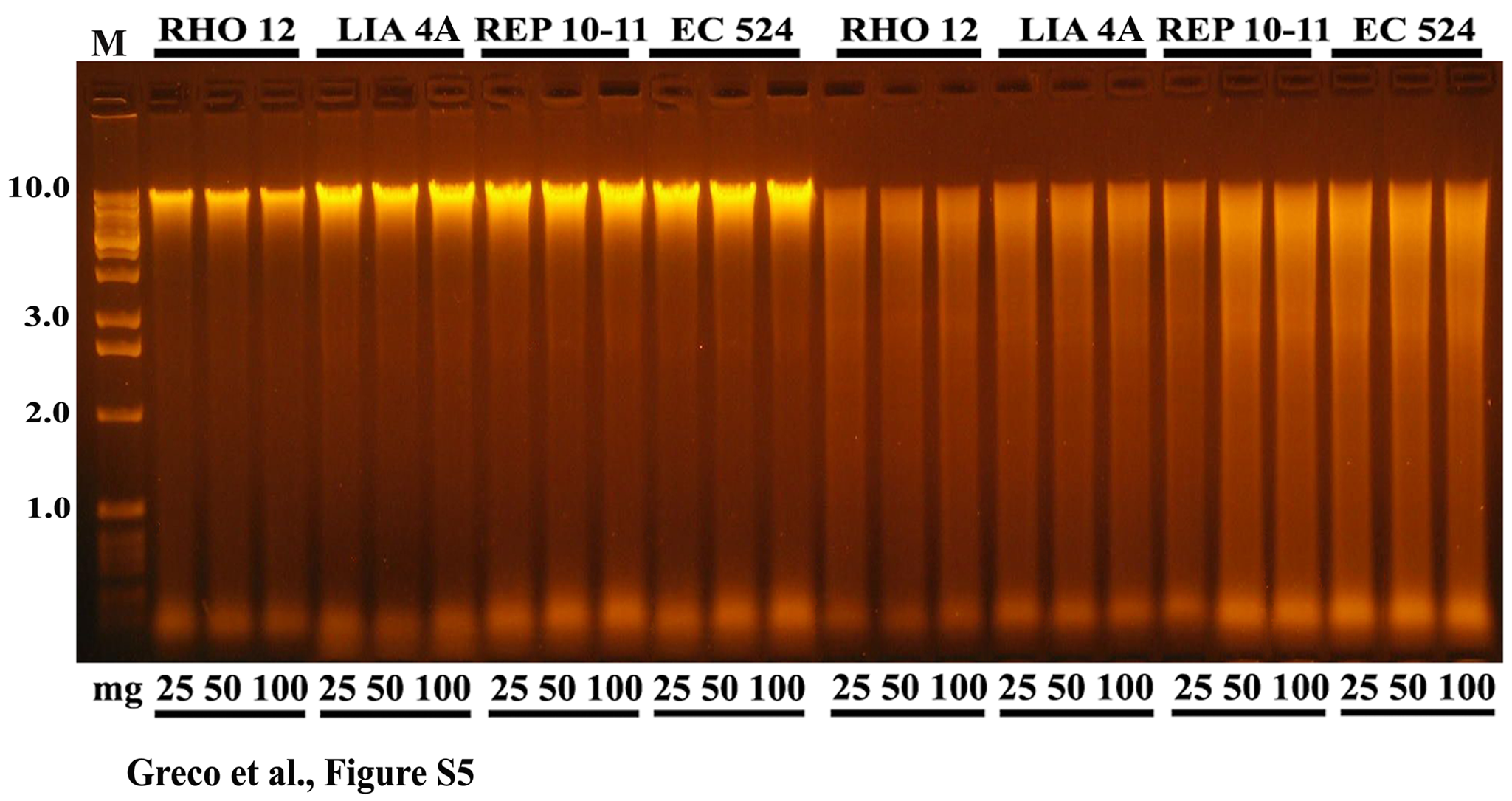

Supplement: Figure S5 — Comparison of undigested and Eco RV digested DNA. Genomic DNA (10 µg) of E. siliculosus strains (RHO12, LIA4A, REP10–11, EC524 from 25, 50 and 100 mg biomass) was digested with EcoRV enzyme (60 units in 200 µl at 37°C, over night) followed by electroforesis on 0.8% agarose gel. The undigested DNA was incubated under the same conditions but without EcoRV enzyme. M: 100 bp ladder. (TIF) [file pone.0096470.s005.tif]
