# Supplementary material for: A Simple and Effective Method for High Quality Co-Extraction of Genomic DNA and Total RNA from Low Biomass Ectocarpus siliculosus, the Model Brown Alga
Source: PLoS One. 2014 May 27;9(5):e96470. doi: 10.1371/journal.pone.0096470 (PMC4035266; doi:10.1371/journal.pone.0096470)
Supplement: Table S1 — Extraction Buffer (EB) guideline. (DOC) [file pone.0096470.s006.doc]

**Greco et al., Table S1**

| **Extraction Buffer (pH 9.5)** | **N° Samples** | **1M**  **Tris-HCl** | **10% Sarkosyl** | **5M NaCl** | **Nuclease-free water** | **1M DTT** |
| --- | --- | --- | --- | --- | --- | --- |
| 10 ml | 6 | 1 mL | 1 mL | 0.3 mL | 7.65 mL | 50 µL |
| 25 ml | 16 | 2.5 mL | 2.5 mL | 0.75 mL | 19.125 mL | 125 µL |
| 50 ml | 32 | 5 mL | 5 mL | 1.5 mL | 38.25 mL | 250 µL |
