# Supplementary material for: A Simple and Effective Method for High Quality Co-Extraction of Genomic DNA and Total RNA from Low Biomass Ectocarpus siliculosus, the Model Brown Alga
Source: PLoS One. 2014 May 27;9(5):e96470. doi: 10.1371/journal.pone.0096470 (PMC4035266; doi:10.1371/journal.pone.0096470)
Supplement: Table S2 — Reagent used to remove contaminants. (DOC) [file pone.0096470.s007.doc]

**Greco et al., Table S2**

| 1V (Volume) Supernatant | e.g. 1.5mL | e.g. 1.4mL | e.g. 1.3mL |
| --- | --- | --- | --- |
| 1/9 V of Absolute Ethanol (pre-cooled) | 166 µL | 155 µL | 144 µL |
| 1/4 V of 3M Potassium Acetate, (4.8 pH) (pre-cooled) | 375 µL | 350 µL | 325 µL |
