# Supplementary material for: A Simple and Effective Method for High Quality Co-Extraction of Genomic DNA and Total RNA from Low Biomass Ectocarpus siliculosus, the Model Brown Alga
Source: PLoS One. 2014 May 27;9(5):e96470. doi: 10.1371/journal.pone.0096470 (PMC4035266; doi:10.1371/journal.pone.0096470)
Supplement: Table S3 — Comparisons of mean values of pure DNA yield and purity between strains isolated from polluted sites (REP10.11, EC524) and those from pristine sites (LIA4A, RHO12). Strains collected from pristine sites exhibit a higher quantity of nucleic acids extracted compared to those from polluted sites. Total amounts of nucleic acids (µg) were calculated in a final volume of 40 µL (a). Data are reported as means ± SE from five independent nucleic acid extractions. Different letters in the DNA yield column represent significant differences according to one-way ANOVA and post-hoc Tukey Test at 95% confidence interval. (DOC) [file pone.0096470.s008.doc]

**Greco et al., Table S3**

| **Strain** | **Starting Material Weight (mg fresh tissue)** | **A_260/280_** | **A_260/230_** | **DNA conc. (ng/µl)** | **Total DNA (µg) ^(a)^** | **DNA Yield (µg/mg)** |
| --- | --- | --- | --- | --- | --- | --- |
| **Mean Value Polluted Sites** | **25** | **1.98 ± 0.017** | **1.97 ± 0.078** | **114.7 ± 8,49** | **4.59 ± 0.34** | **0.183 ± 0.013^a^** |
|  | **50** | **1.91 ± 0.007** | **1.83 ± 0.056** | **197.4 ± 10.3** | **7.89 ± 0.41** | **0.158 ± 0.008^a^** |
|  | **100** | **1.85 ± 0.007** | **1.75 ± 0.036** | **352.1 ± 13.0** | **14.06 ± 0.52** | **0.141 ± 0.005^a^** |
| **Mean Value Pristine Sites** | **25** | **1.87 ± 0.02** | **1.69 ± 0.02** | **241.1 ± 13.7** | **9.64 ± 0.54** | **0.385 ± 0.022^b^** |
|  | **50** | **1.84 ± 0.013** | **1.67 ± 0.023** | **332.1 ± 11.5** | **13.27 ± 0.45** | **0.265 ± 0.009^c^** |
|  | **100** | **1.81 ± 0.013** | **1.67 ± 0.022** | **522.2 ± 53.8** | **20.87 ± 2.16** | **0.208 ± 0.021^c^** |
