# Supplementary material for: A Simple and Effective Method for High Quality Co-Extraction of Genomic DNA and Total RNA from Low Biomass Ectocarpus siliculosus, the Model Brown Alga
Source: PLoS One. 2014 May 27;9(5):e96470. doi: 10.1371/journal.pone.0096470 (PMC4035266; doi:10.1371/journal.pone.0096470)
Supplement: Table S5 — Mean nucleic acids yield reduction (%) obtained with the old method. A differential decrease in the quantity of nucleic acids was recorded for all strains when the old method [47] was used compared with the new one. (DOC) [file pone.0096470.s010.doc]

**Greco et al., Table S5**

|  | **DNA** | **RNA** |
| --- | --- | --- |
| **REP 10-11** | 65% | 64% |
| **EC 524** | 43% | 41.6% |
| **RHO 12** | 40% | 32% |
| **LIA 4A** | 16% | 9.6% |
