# Supplementary material for: A Simple and Effective Method for High Quality Co-Extraction of Genomic DNA and Total RNA from Low Biomass Ectocarpus siliculosus, the Model Brown Alga
Source: PLoS One. 2014 May 27;9(5):e96470. doi: 10.1371/journal.pone.0096470 (PMC4035266; doi:10.1371/journal.pone.0096470)
Supplement: File S1 — List of consumables, solutions and reagents, equipment as well as a guideline of nucleic acids extraction. (DOC) [file pone.0096470.s011.doc]

**Consumables**

- 1.5 - 2.0 mL microcentrifuge tubes DNase- and RNase-free (both tubes have ultrasmooth conical bottoms to allow 100% visibility) (e.g. Axygen Microcentrifuge Tubes, Axygen Scientific, Cat. No MCT-150-B);
- 15 mL Corning Centrifuge tubes DNase- and RNase-free (e.g. GeneMate, Cat. No C-3394-1);
- 3 mm solid-glass beads (e.g. Sigma Aldrich, Cat. No Z143944);
- Laboratory goggles;
- Micropipette (e.g. Gilson or Eppendorf);
- Pestle Mixer RNase-free, used for grinding soft tissue in microcentrifuge tubes (e.g. Krackeler Scientific, Inc. Cat. No 186-749520-0090).

NOTE: If using alternative plastic-ware, make certain it is resistant to phenol and chloroform.

**Solutions and reagents**:

- RNA Extraction Buffer (EB): 100 mM Tris-HCl, pH 9.5; 150 mM NaCl; 1.0% sarkosyl. Add 5 mM DTT before use;
- 100% and 75% (v/v) ethanol made with DEPC-treated double-distilled (dd) H2O; (Sigma-Aldrich, Cat. No 02860);
- 1 M Dithiothreitol (DTT) (Sigma-Aldrich, Cat. No 646563);
- 2-mercaptoethanol (Sigma-Aldrich, Cat. No M3148);
- Chloroform (Sigma-Aldrich, Cat. No 25690);
- DEPC-treated 3 M sodium acetate (pH 5.2) (**Sigma-Aldrich,** Cat. No **S2889)**;
- DEPC-treated 3 M potassium acetate (pH 4.8) (**Sigma-Aldrich,** Cat. No **P1190)**;
- Diethylprocarbonate (DEPC) (Sigma-Aldrich, Cat. No D5758). CAUTION:DEPC is a carcinogen, work under fume hood;
- DNase I recombinant, RNase freee enzyme (Roche Diagnostic, Cat. No 04716728001);
- Isoamyl alcohol (3 Methyl-1 Butanol, 99%), (Sigma-Aldrich, Cat. No W205710);
- Isopropanol (2-propanol) (Sigma-Aldrich, Cat. No I9516);
- Liquid nitrogen;
- Phenol solution pH 8.0 (Sigma-Aldrich, Cat. No P4557);
- Ribonuclease A enzyme (Sigma-Aldrich, Cat. No R4875);
- RNAse-free water. Add 1 ml l^-1^ DEPC, shake the solution and incubate overnight under a fume hood. Autoclave properly to inactivate remaining DEPC.

**For Agarose gel electrophoresis analysis**

- Agarose (Roche Cat. No 11388991001);
- Ethidium bromide (Sigma-Aldrich, Cat. No E7637);
- TBE 5X concentrate (Sigma-Aldrich, Cat. No 93306). TBE was prepared in RNase-free water to reach the TBE 0.5X working solution;
- RNA ladder High Range (Fermentas, Cat. No SM0423);
- DNA ladder Gene Ruler ™, 100 bp and 1kb (Fermentas, Cat. No SM0241, SM0312).

**Equipment**

- Autoclave;
- Benchtop laboratory shaker (e.g. INNOVA® 2000 – 2050);
- DNA electrophoresis equipment;
- Eppendorf Minispin (e.g. Eppendorf Plus w Rotor Nice);
- Fume cupboard for handling phenol, chloroform, DTT and 2-mercaptoethanol;
- NanoDrop ND-1000 Spectrophotometer;
- Refrigerated laboratory centrifuge (e.g. Eppendorf 5430 R);
- Standard laboratory instruments (beakers, cylinders, flasks, magnetic stirrer, tweezers etc);
- Thermocycler (e.g. Eppendorf);
- Thermomixer (e.g. Eppendorf);
- Vortex (e.g. Genie® 2 (S-7350-1));

**Note:** Glassware and pestles were cleaned with detergent, filled with 0.1% DEPC, incubated at 37°C overnight, and then autoclaved for 30 minutes.

**Guidelines of nucleic acids extraction**

**Before starting**

Ensure you have sufficient volume of stock solutions: 1M Tris-HCl (pH 9.5), 1M DTT, 3M potassium acetate (pH 4.8), 3M sodium acetate (pH 5.2), 5M NaCl, 10% sarkosyl, 100% ethanol.

Keep isopropanol, 75% and 100% ethanol at -20ºC. Solutions 1M Tris-HCl (pH 9.5), 3M potassium acetate (pH 4.8), 3M sodium acetate (pH 5.2), 5M NaCl, (24:1, v/v) chloroform:isoamyl alcohol, phenol, 10% sarkosyl and 1M DTT must be kept at 4ºC.

**WARNING:** For DNA extraction, use sterilized tips, tubes and solutions; For RNA extraction, RNase-free tips, tubes and any other consumables must be used. Moreover, use RNase free/DEPC treated solution throughout. All the equipment should be well cleaned, a surface decontaminant solution that destroys RNases can be used; they are ideal for cleaning work surfaces, pipettors, and equipment that must be RNase-free (e.g. Ambion® RNaseZap®).

**WARNING:** All extraction steps, including centrifugation, must be carried out under cold conditions (e.g. on ice or in a cold-room). Use a fume hood and wear personal protective equipment when handling 2-mercaptoethanol, DTT phenol and chloroform. Always wear clean gloves and change gloves frequently.

**WARNING:** During tissue harvesting do not overfill tubes and ensure algal material is free of surface water for optimal tissue homogenisation. To obtain the best quality nucleic acids it is essential that harvested material is frozen rapidly and that the material is not allowed to thaw. At this stage, material can be stored at -80°C for several weeks before extraction.

**WARNING:** Change gloves immediately if you spill chloroform on them. Avoid dripping chloroform into the tubes; due to it low viscosity chloroform drips out of the tip and could make the label peel off of the tube.

**WARNING:** Remove the aqueous phase carefully; avoid jerky movements to prevent re-mixing with interphase layer.

**NOTE:** The nucleic acid pellet is considered dry when no further drops or liquid is observable; it typically occurs within 30-45 minutes. Be careful not to lose the pellet which is securely attached to the tube since rough handling can dislodge it. Moreover, if the pellet is dried upside down for too long, it will fall out.
